# Supplementary material for: Deep learning-based assessment of pulp involvement in primary molars using YOLO v8
Source: PLOS Digit Health. 2025 Apr 8;4(4):e0000816. doi: 10.1371/journal.pdig.0000816 (PMC11977986; doi:10.1371/journal.pdig.0000816)
Supplement: Attachment — Submitted filename: Response to Reviewers.docx [file pdig.0000816.s002.docx]

Dear Editor-in-Chief, and

Dear Section Editor

We appreciate the time and effort the esteemed reviewers have dedicated to our manuscript and for providing valuable feedback.

In this letter, we have included detailed responses to the comments and outlined how we incorporated their recommendations into the revised manuscript. Below, you will find our point-by-point responses to each comment:

1. We ask that a manuscript source file is provided at Revision. Please upload your manuscript file as a .doc, .docx, .rtf or .tex.

The manuscript source file has been uploaded as requested.

2. In the online submission form, you indicated that The datasets used and analyzed during the current study are available from the corresponding author upon reasonable request. All data generated or analyzed during this study are included in this published article.

All PLOS journals now require all data underlying the findings described in their manuscript to be freely available to other researchers, either

1. In a public repository,

2. Within the manuscript itself, or

3. Uploaded as supplementary information.

This policy applies to all data except where public deposition would breach compliance with the protocol approved by your research ethics board. If your data cannot be made publicly available for ethical or legal reasons (e.g., public availability would compromise patient privacy), please explain your reasons by return email and your exemption request will be escalated to the editor for approval. Your exemption request will be handled independently and will not hold up the peer review process, but will need to be resolved should your manuscript be accepted for publication. One of the Editorial team will then be in touch if there are any issues.

Data Availability Statement: The data supporting this study's findings are available from the University of Alberta. However, restrictions apply to the availability of these data, which were used under license for the current study and are not publicly available. The data can be requested via email at [dentrsch@ualberta.ca](mailto:dentrsch@ualberta.ca), subject to request and with permission from the University of Alberta.

3. Reviewer #1: Comments

Main Claims and Significance

The manuscript claims to have developed a machine learning model using the YOLOv8 architecture to diagnose pulp involvement in primary molars through bitewing radiographs. This is significant for pediatric dentistry as it offers a potentially more accurate and efficient method for assessing dental caries, which is a prevalent issue among children.

Novelty and Contextual Placement

The use of the YOLOv8 model is relatively novel in this specific application, as previous studies have utilized older versions of YOLO for different dental diagnostics. The manuscript should better contextualize its claims by comparing them with existing literature, particularly studies that have used similar machine learning approaches in dental radiology for assessing pulp involvement. This would help in establishing the originality and contribution of the current study.

This is a relatively new field within the application of ML in endodontics and pediatric dentistry. To the best of our knowledge, only three articles have been published on this topic to date.

We agree that contextualizing our study with relevant literature would strengthen our manuscript. To address this, we have revised the discussion section to include comparisons with three key studies in this field:

I) **Ma et al. (2024)**: This study was already referenced in the original manuscript.
II) **Wang et al 2023:**

Wang et al. evaluated ML-based predictions of pulp exposure in permanent teeth using periapical radiographic images. Their methodology closely resembled ours, particularly in the selection of reference standard and case-labeling processes. However, their study focused on permanent teeth and periapical images, whereas ours concentrated on primary teeth and bitewing radiographs. Additionally, while Wang et al. employed three ML models (VGG, ResNet, and DenseNet), we adapted the YOLOv8 architecture for classification. DenseNet achieved the best performance in their study (AUC = 0.97), followed by ResNet (AUC = 0.89), VGG (AUC = 0.78), and senior dentist (AUC = 0.87). Their reported accuracy levels for the three trained models ranged between 75% and 85%, which are comparable to ours (78.7%–87%).

**Reference**: Wang L, Wu F, Xiao M, et al. *Prediction of pulp exposure risk of carious pulpitis based on deep learning.* Hua Xi Kou Qiang Yi Xue Za Zhi. 2023 Apr 1;41(2):218-224. [https://pmc.ncbi.nlm.nih.gov/articles/PMC10427250](https://pmc.ncbi.nlm.nih.gov/articles/PMC10427250/)

III) **Ramezanzade et al. (2024)**:
     Ramezanzade et al. compared the effect of providing AI-based radiographic information versus standard radiographic and clinical information on dental students' ability to predict pulp exposure. While their study used bitewing radiographs similar to ours, the primary goal was to assess the impact of AI assistance through the application of convolutional neural networks (CNN) based on ResNet-50 architecture on students' diagnostic capabilities. Their findings showed that AI outperformed the students, the participants only benefited "slightly" when provided with AI predictions. Notably, the authors emphasized the need for more explainable AI systems to address real-world clinical complexities, including factors such as provider experience and patient expectations.

**Reference**: Ramezanzade S, Dascalu TL, Ibragimov B, et al. *Prediction of pulp exposure before caries excavation using artificial intelligence: Deep learning-based image data versus standard dental radiographs.* J Dent. 2024;138:104732. <https://doi.org/10.1016/j.jdent.2023.104732>

4. Data and Analysis Support
   The data and analyses appear to support the claims, with the model achieving notable accuracy rates for both upper and lower primary molars. However, the study could benefit from additional validation, such as comparisons with diagnoses from dentists.

In this study, we used the condition of pulp involvement after cavity preparation as the gold standard, and the performance metrics presented reflect the evaluation of the model's performance against this ground truth.

Comparing the model’s performance with clinicians’ diagnostic performance was beyond the scope of this study, as the focus was solely on developing and evaluating the model against a definitive clinical standard. However, we recognize the value of such comparisons and plan to address this in future studies to further validate the model's applicability in clinical settings.

5. Reviewer #1: 1. Comparison with reference standard model/other models: The study can benefit by including either:
   a. an objective comparison with an existing data science model predicting the same outcome.
   b. a clinical score used in clinical settings objectively using a numeric value or a metric (if outcome of score and algorithm is same).
   c. an objective comparison with results obtained by a group of dentists.
   d. an objective comparison based on clinical experience/judgement.

In this study, the condition of pulp involvement after cavity preparation was used as the gold standard, and the performance metrics presented reflect the model’s evaluation against this definitive clinical standard.

While comparisons with existing data science models, clinical scoring systems, and clinicians’ diagnostic performance are valuable, they were beyond the scope of this study, as the primary objective was to establish and validate the model based on a clinically confirmed reference standard.

We acknowledge the importance of these broader comparisons for enhancing clinical relevance and plan to address them in future studies. These efforts will include validating the model against existing data science models, incorporating clinical scoring systems, and comparing its performance with diverse groups of clinicians to further assess its applicability in real-world dental practice.

6. Reviewer #1: 2. Reproducibility: The manuscript should provide more detailed descriptions of the methodology, including data preprocessing and model training steps, to ensure reproducibility. A GitHub repository or at least supplementary material should suffice.

We appreciate the reviewer’s emphasis on ensuring reproducibility. To address this, we have provided detailed methodology descriptions, including data preprocessing, model training steps, and evaluation techniques.

These details have been added to the **Methods** section:

“The images were resized to 280x280 pixels (updated to 288x288 for compatibility with the model's stride) and normalized using standard values. The YOLOv8 classification model was trained for 40 epochs using a batch size of 16, AdamW optimizer, and an auto-determined learning rate of 0.000714. Mixed precision was enabled to optimize training performance. Pretrained weights were utilized, with 228 out of 230 items successfully transferred, and training/validation metrics were logged using TensorBoard for transparency.”

Due to university guidelines restricting the public sharing of patient data, we are unable to create a public GitHub repository with the dataset. However, the manuscript provides sufficient details about the model architecture, training process, and evaluation metrics to enable other researchers to replicate our methodology using similar data. Supplementary material, including specific configurations and model details, has been added to further enhance reproducibility.

7. Reviewer #1: 3. Calibration performance of the model: Calibration performance metrics like calibration plot or Brier score should be evaluated.

We appreciate the reviewer’s suggestion regarding the evaluation of calibration performance. YOLOv8 is primarily designed for object detection, but in this study, it was adapted for classification. Although calibration performance metrics like calibration plots or the Brier score are typically used for probabilistic models, YOLOv8 generates confidence scores for its predictions, which can be analyzed for calibration.

In this study, we focused primarily on the model’s classification accuracy and other performance metrics, so calibration metrics were not included. However, we recognize the importance of calibration in assessing the reliability of confidence scores. Future work will explore calibration techniques, such as temperature scaling or isotonic regression, and evaluate calibration metrics (e.g., the Brier score and calibration plots) to further assess the model’s reliability in clinical settings.

8. Reviewer #1: 4. Reporting of parameter estimates: We can also present the initial and final values of weights and biases for each layer of the CNN. This could be done in a tabular format or as part of an appendix to avoid cluttering the main text. Also, a comprehensive description of the model architecture, specifying the number of layers, types of layers (e.g., convolutional, fully connected), and activation functions used would be helpful.

We appreciate the reviewer’s suggestion to include details about the model architecture and parameter estimates. YOLOv8, while primarily designed for object detection, was adapted for this study as a classification model. It includes a combination of convolutional layers, activation functions (e.g., Leaky ReLU), and fully connected layers, depending on the specific architecture and implementation.

To avoid cluttering the main text, a comprehensive description of the model architecture, including the number and types of layers, activation functions, and any relevant modifications made for classification, will be included in an appendix. While presenting initial and final values of weights and biases for each layer is not a standard practice due to the complexity and size of modern neural networks, we will consider providing an overview of key parameter settings and training configurations to enhance transparency.

To address this comment, we have included the explanation below in our **Methods**:

“The model was trained for 40 epochs with an initial image size of 280, which was automatically adjusted to 288 to ensure compatibility with the model's stride. The learning rate was set to 0.0001, determined automatically by the AdamW optimizer used during training. A batch size of 16 was employed, and mixed precision was enabled to optimize computational efficiency. Pretrained weights were utilized for model initialization, with 228 out of 230 items successfully transferred from the pretrained YOLOv8m-cls model. This facilitated better starting performance and improved convergence during training. Additional adjustments, such as updating the image size for stride compatibility, were made automatically by the model's configuration. The entire training process, including the results and model architecture, was logged using TensorBoard, enabling detailed visualization and analysis of the training and validation phases.”

9. Reviewer #1: 5. Model interpretability: Incorporating heat maps could provide insights into the model's decision-making process, enhancing transparency and understanding of the AI's focus areas.

Grad-CAM visualizations were generated to illustrate the model's focus areas during classification for both positive (pulp involved) and negative (pulp not involved) samples, highlighting regions relevant to clinical diagnoses. Following are the Grad-CAM images for reference.

Grad-CAM images:

Pulp not involved

Pulp involved

10. Reviewer #2: The topic was interesting and there is certainly value in the area of dental education, especially in a movement towards selective caries removal and vital pulp therapy. A couple of comments:

(1) The title and thrust of the manuscript implies that radiographic findings alone dictate decision-making in treatments of primary pulp tissue. While the authors do mention the importance of clinical findings when generate treatment plans, it is unclear how this deep learning-based assessment will account for the histologic status of the pulp tissue and the clinical findings. Consider rewording the title.

(2) The authors do a good job understanding and describing the limitations of the study. There would be value in a follow-up study that looks at the long-term outcomes of the teeth that were treated conservatively, to understand if the model can reliably predict long-term outcomes of various treatment modalities.

(1) **Regarding the title and the inclusion of clinical findings**:
We acknowledge the concern regarding the potential implication that radiographic findings alone dictate treatment decisions. As emphasized in the manuscript, the importance of clinical findings in generating treatment plans is indeed highlighted. However, we recognize that our deep learning-based assessment does not currently account for the histologic status of the pulp tissue or clinical findings. To clarify this, we have made it clear in the manuscript that **clinical data was not incorporated in the training of the model**. Since the study emphasized **bitewing radiographs** (as reflected in the title), we opted not to include clinical data for training the model. Given the constraints of word count and to avoid altering the focus of the manuscript, we would like to retain the original title, as it accurately reflects the scope of the study.

(2) **Regarding the suggestion for a follow-up study on long-term outcomes**:
We appreciate the reviewer’s suggestion regarding a follow-up study to assess the long-term outcomes of conservatively treated teeth. We agree that it would be valuable to evaluate whether the model can reliably predict long-term treatment outcomes. We acknowledge that this is an important area for future research and will consider it in the direction of subsequent studies.

11. Reviewer #3: In diagnosing the relationship between caries in primary molars and the pulp in children using the bitewing method, if AI support constructed in this study can be obtained, it may lead to an improvement in prognosis. However, one point of caution in building the AI is the labeling accuracy during dataset construction. Was the labeling done by pediatric dentistry specialists? How was the judgment made by referring to medical information, electronic medical records, and clinical information? What were the exclusion criteria for each case? Furthermore, is there any variation in human judgment? Medical information, electronic medical records, and clinical information may not always accurately reflect phenomena or prognosis; sometimes, they are recorded for convenience in calculating treatment costs. From a dental perspective, referring to medical information, electronic medical records, and clinical information, the influence of the initial diagnosis at the time of the first consultation cannot be ignored. In other words, the diagnosis of caries is not standardized by dentists, and in cases where there is no problem with the pulp, pulp treatment might have been unnecessarily performed, without any backup support. Therefore, it is necessary to address the issue of reliability concerning the evidence obtained from the retrospective study in this research.

In this study, we used the presence of pulp involvement after cavity preparation as the gold standard. The performance metrics presented reflect the model's evaluation against this ground truth. The images used in this study were labeled based on definitive diagnoses made in the clinic. These diagnoses were established through direct examination of the pulp exposure after cavity preparation and caries removal, as documented in dental charts, not solely relying on treatment cost codes.

This crucial information has now been included in the “Methods” section to provide clarity and transparency regarding the labelling process and its role in training the model.

12. **Reproducibility:** To enhance the reproducibility of your results, we recommend that authors of applicable studies deposit laboratory protocols in [protocols.io](http://protocols.io/), where a protocol can be assigned its own identifier (DOI) such that it can be cited independently in the future.

Thank you for the suggestion. We acknowledge the importance of enhancing reproducibility, however, our study is not laboratory-based and does not involve specific experimental protocols.
